# DNA methylation-based sex classifier to predict sex and identify sex chromosome aneuploidy

**DOI:** 10.1186/s12864-021-07675-2

**Published:** 2021-06-28

**Authors:** Yucheng Wang, Eilis Hannon, Olivia A. Grant, Tyler J. Gorrie-Stone, Meena Kumari, Jonathan Mill, Xiaojun Zhai, Klaus D. McDonald-Maier, Leonard C. Schalkwyk

**Affiliations:** 1 School of Computer Science and Electronic Engineering, University of Essex, Wivenhoe Park, Colchester, UK; 2 Medical School, University of Exeter, Barrack Road, Exeter, UK; 3 School of Biological Sciences, University of Essex, Wivenhoe Park, Colchester, UK; 4 Diamond Light Source Ltd., Harwell Science & Innovation Campus, Oxfordshire, UK; 5 Institute for Social and Economic Research, University of Essex, Wivenhoe Park, Colchester, UK

**Keywords:** DNA methylation, Sex prediction, Aneuploidy

## Abstract

**Background:**

Sex is an important covariate of epigenome-wide association studies due to its strong influence on DNA methylation patterns across numerous genomic positions. Nevertheless, many samples on the Gene Expression Omnibus (GEO) frequently lack a sex annotation or are incorrectly labelled. Considering the influence that sex imposes on DNA methylation patterns, it is necessary to ensure that methods for filtering poor samples and checking of sex assignment are accurate and widely applicable.

**Results:**

Here we presented a novel method to predict sex using only DNA methylation beta values, which can be readily applied to almost all DNA methylation datasets of different formats (raw IDATs or text files with only signal intensities) uploaded to GEO. We identified 4345 significantly (*p*<0.01) sex-associated CpG sites present on both 450K and EPIC arrays, and constructed a sex classifier based on the two first principal components of the DNA methylation data of sex-associated probes mapped on sex chromosomes. The proposed method is constructed using whole blood samples and exhibits good performance across a wide range of tissues. We further demonstrated that our method can be used to identify samples with sex chromosome aneuploidy, this function is validated by five Turner syndrome cases and one Klinefelter syndrome case.

**Conclusions:**

This proposed sex classifier not only can be used for sex predictions but also applied to identify samples with sex chromosome aneuploidy, and it is freely and easily accessible by calling the ‘estimateSex’ function from the newest *wateRmelon* Bioconductor package (https://github.com/schalkwyk/wateRmelon).

**Supplementary Information:**

The online version contains supplementary material available at (10.1186/s12864-021-07675-2).

## Background

DNA methylation is one of the most-studied epigenetic modifications, which typically occurs in the context of a cytosine-guanine dinucleotide motif (CpG) [1]. DNA methylation plays important roles in the stability and regulation of gene expression in the development and maintenance of cellular identity [2]. The dynamic process of DNA methylation and the plasticity of the DNA methylation landscape make genes responsive to the changes of environmental conditions. Several health and lifestyle factors have been found to be associated with DNA methylation signatures, including childhood disease, tobacco smoke, drug use and poor nutrition [3–5].

Genome-wide analysis of DNA methylation has now become popular and is growing rapidly, owing to array-based profiling technologies. The two most widely used microarray platforms, Infinium HumanMethylation450 BeadChip (450K) [6] and Infinium MethylationEPIC BeadChip (EPIC) [7], offer broad coverage and precise quantification of DNA methylation levels at roughly 480,000 and 860,000 CpG sites respectively.

Epigenome-wide Association Studies (EWAS) are a powerful way to study the relationships between epigenetic variation and human diseases [8]. Apart from sex chromosomes, thousands of CpG sites on autosomes also show very different DNA methylation patterns between males and females [9, 10]. As a result of this, sex has been considered an important co-variate, when undertaking methylation and phenotype association studies.

Many researchers have submitted their methylation microarray datasets to the Gene Expression Omnibus (GEO). Currently, there are over 100,000 HM450k samples and over 18,000 EPIC samples which are publicly available. Most of these have phenotype annotations accompanying them, thus they can be used by other researchers to perform meta-analyses or as independent references to validate their hypothesis. However, many mismatches have been found between annotations and samples, Toker et al. discovered widespread mislabelling in transcriptomics datasets of GEO [11], Heiss et al. found 25% of the datasets they studied contained sex-mismatched samples, particularly in three datasets, more than 30% of the samples were identified as being mislabelled [12]. A large portion of these discrepancies may stem from data entry errors. Researchers should deal with these sex-mismatched samples carefully; the safest way is to remove them directly before downstream analysis.

McCarthy and colleagues performed meta-analysis of sex-specific methylation patterns and demonstrated that the first two principal components of X chromosome methylation data on 27k arrays can differentiate between sexes [13]. Currently, there are several methods which can be used to predict the sex of samples from DNA methylation data. The ‘getSex’ function of *minfi* package estimate sex based on the median values of measurements on the X and Y chromosomes respectively [14]; the ‘estimateSex’ method of *sEst* package groups beta values and detection *p*-values of probes mapped on sex chromosomes into different intervals and achieved sex prediction by looking at the different distribution patterns of these intervals from two sexes. [15]; The ‘check _sex’ method within the *ewastools* package predict sex based on normalized average signal intensity values on the sex chromosomes [12].

In this paper, we propose a novel method to predict the sex of samples using solely DNA methylation beta values. We identify a set of significant sex-associated CpG sites, and perform principal component analysis (PCA) on these sites to obtain a sex classifier, and evaluate our method’s performance across a wide range of human tissues. The proposed sex classifier allows users to attribute sex to un-annotated samples on public databases, and also identify samples with sex aneuploidy.

## Results

### Identifying sex-associated CpG loci

To make our method compatible with both 450K and EPIC, we only included 453,152 probes that are present on both arrays. Two-sample *T*-tests were applied to GSE105018 [16] to identify differentially methylated CpG sites between sexes, after Bonferroni multiple comparison correction, those with *p*-value less than 0.01 and absolute beta value difference between sexes greater than 0.2 were selected as the most significant sex associated CpG sites. As a result of this, we obtain 4345 significantly sex-associated sites. In this study we have chosen a relatively strict threshold, as we aim to capture those most robust features which methylate differently and consistently between the two sex groups across various datasets. As expected, most of the sex-associated sites belong to sex chromosomes, with the majority (4047, 93%)located on the X chromosome (ChrX), and with a total of 284 (6.5%) CpG sites located on the Y chromosome (ChrY) Additional file 1.

**Fig. 1:**
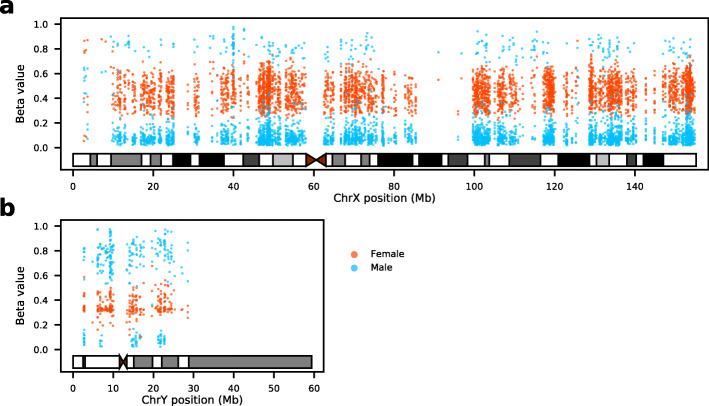
Females and males exhibit distinct methylation patterns at sex-associated CpG sites on the two sex chromosomes **a** The X chromosome: most sex-associated CpG sites from females have beta values range between 0.2 and 0.8; most of these sites from males are less methylated (beta values less than 0.2). **b** The Y chromosome: the identified sex-associated CpG sites of males are highly methylated with beta values greater than 0.6 whereas females exhibited low methylation signals

As shown in Fig. 1a, these sex-associated CpG sites on ChrX are distributed throughout the whole chromosome, and with most of them (3781, 93.4%) associated with higher methylation levels in females compared to males, this is mainly because one X chromosome of the female is inactivated and highly methylated. However, we also observed a small portion of CpG sites (266, 6.6%) on ChrX that have higher methylation levels in males compared to females, this could attribute to the facts that around 15% of X-chromosome genes often escape from XCI and another fifteen percentage shows variable degree of ‘escape’ [17]. For example, four out of the 266 probes mapped to *Xist* which is an escape gene with known exclusive expression from the inactivated X chromosome [17].

Among the 284 sex-associated CpG sites on ChrY, 211 CpG sites have higher methylation levels in male samples (Fig. 1b). Females do not carry Y chromosomes, thus most of the intensity signals of ChrY we observed from females are may due to background noise and non-specific hybridisation, nevertheless, the mean raw signal intensities of the 284 probes in females are only around 11% of that in males. Interestingly, 70 of the 284 probes are on McCartney’s list of 67,609 potential non-specific probes of EPIC array [18], however, 69 of them are hypermethylated in males (mean=0.73, sd=0.11), while hypomethylated in females (mean=0.35, sd=0.07). The raw signal intensities of the 70 probes in females are also only around 10% of that in males, suggesting they were less affected by the non-specific hybridisation issue.

### Sex classifier based on sex-associated CpG sites

**Fig. 2:**
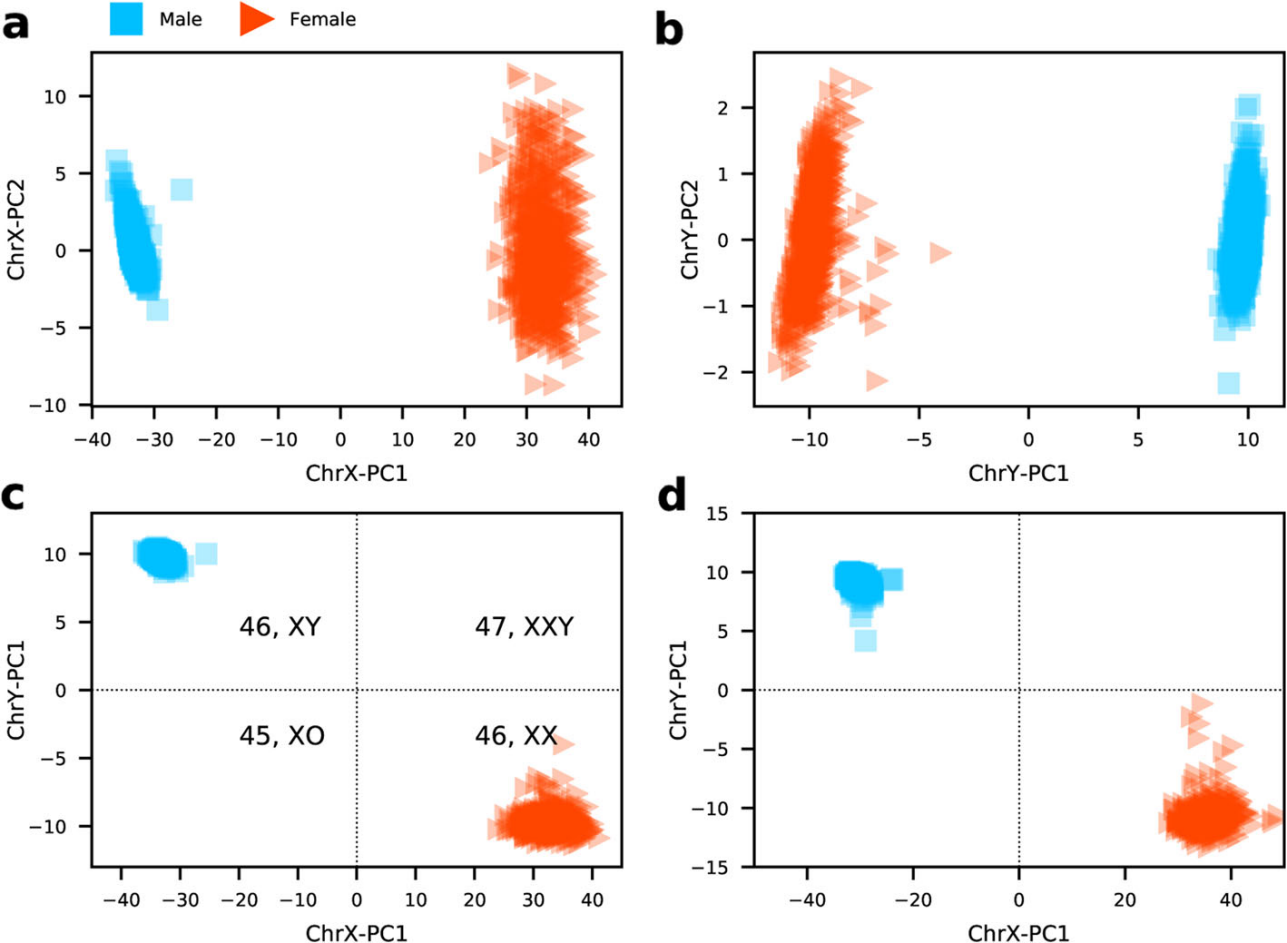
A sex classifier is constructed by applying two PCAs on two sex chromosomes separately. **a** The first two components on ChrX. **b** The first two components on ChrY. Results of **c** training set and **d** validation set produced by the sex classifier, all samples are classified into four categories: 46XY, 46XX, 47XXY, and 45XO

Since we have obtained a large group of CpG sites which show a significant difference (*p*<0.01) in methylation levels between males and females, we are able to construct a sex classifier. To begin with, the DNA methylation values of the 4047 sex-associated CpG sites on ChrX from the same training samples are processed using PCA. PCA takes a linear approach to generate reduced dimensions by maximizing the captured residual variance in each further dimension [19]. As shown in Fig. 2a, the first principal component, which explained 98% of total variance, has captured the most sex differences among the all training samples. Thus, we could use this first component to separate samples into two categories: 1) with two copies of X chromosomes and 2) with only one copy of X chromosome.

Similarly, a PCA is performed using the 284 CpG sites of ChrY, and as that of ChrX, the first principal component accounted for the most variances can make a good separation between male and female samples (Fig. 2b). As the result of this, the first component can be used to divide samples into two categories: 1) with Y and 2) without Y.

Finally, the two first principal components of the two PCAs which both explained the most sex differences are utilized to build the sex classifier. Normal females have two copies of X chromosomes and normal males have one copy of X chromosome and one copy of Y chromosome. By our sex classifer, male samples with 46,XY should locate in the top left area and female samples with 46,XX should distribute at the bottom right area (Fig. 2c). It is reasonable to suggest that this model can be applied to identify samples with sex aneuploidy: samples with 45,XO will be placed at the bottom left corner, and samples with 47,XXY should be distributed at the top right corner.

### Comparison with other tools

**Fig. 3:**
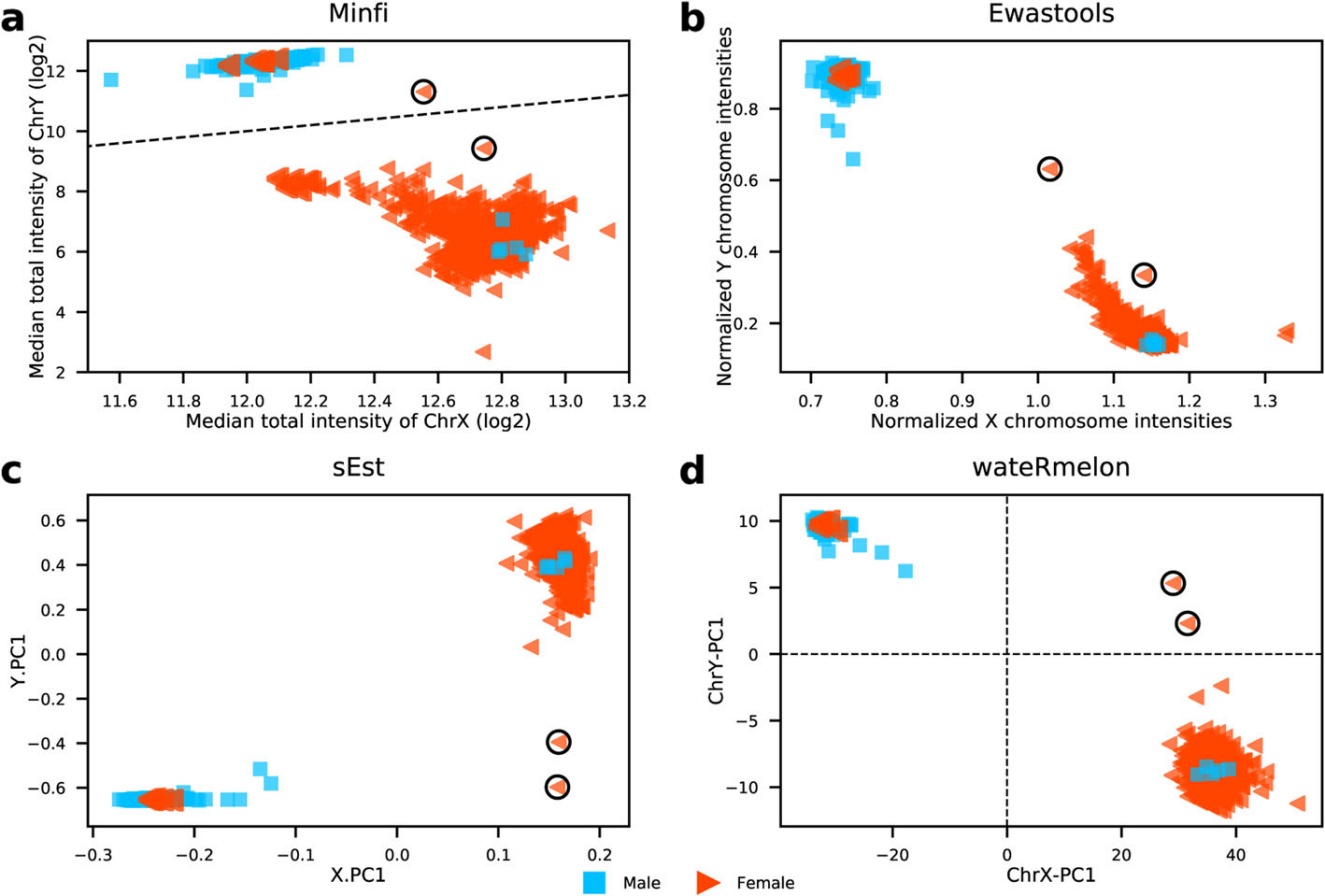
Comparisons of sex prediction ability between four tools. **a** minfi, **b** ewastools, **c** sEst, **d** our classifier in wateRmelon. Two outlier samples are marked by black circles, blue square represents male and red triangle denotes female

To compare the proposed sex classifier with three other existing sex prediction classifiers for DNA methylation microarray data taken from the R packages (see Table 1), *minfi* [14], *ewastools* [12] and *sEst* [15], we take GSE51032 [20] as a benchmark dataset, as it was used in developing *ewastools* and *sEst*. GSE51032 includes 857 samples (188 men and 657 women) and their source tissue are all from buffy coat. Figure 3 shows the results generated by the four methods, as we can see, there are eight samples (four males and four females) displaying mismatches between predicted sex and labelled sex, and the mismatches are consistent in the results from four methods, thus we have high confidence that the eight samples are mislabelled. Two samples (marked by black circles) are identified by our classifier as 47,XXY, *sEst* also identified the two outliers. However, only one of the two samples appears as an outlier from *minfi* and *ewastools*, and the other one stays close with the main male cluster.

**Table 1:** Summary of four sex prediction tools for DNA methylation samples

Package	Function name	Input requirements	Mechanism	Performance on clustering females and males	SCA detection
Minfi	getSex	IDATs	Compare the log2 transformed median total intensity of probes mapped on ChrX and ChrY.	Good in clustering males and less well in clustering females	Not provided
Ewastools	check_sex	IDATs	Compare the normalized average signal intensity of probes mapped on sex chromosomes	Excellent in clustering males and good in clustering females	Not provided
sEst	estimateSex	Beta values and detection *p*-values	Group beta values and detection *p*-values into defined intervals and PCAs on the distribution patterns of these intervals.	Excellent in clustering males and females	Proposed but not validated
WaterRmelon	estimateSex	Beta values (which can be easily generated from signal intensity text files or IDATs)	PCAs on beta values of sex differently methylated CpGs on ChrX and ChrY separately.	Excellent in clustering males and females	Proposed and validated by five Turner syndrome cases and one Klinefelter syndrome case

In general, all four methods show good performance in clustering male samples, however the method from *minfi* performs much poorer in clustering female samples compare to the other three tools, as some females are not distinguishable from males along the x-axis. The female cluster produced by *ewastools* exhibits long tail towards the male cluster; the sex prediction tools in *minfi* and *ewastools* are both based on signal intensity therefore they produce more similar results than the other two tools. Our sex classifier and the method from *sEst* are both beta value based, although the two methods utilised beta values very differently and *sEst* requires detection *p*-values, the patterns of their results are similar. It should be noted, detection *p*-values are used as an index of usability for each probe but are not well defined. It is implemented as a test for signal intensity above background level in the proprietary GenomeStudio software, the detection *p*-values calculated by the minfi package are better documented but not equivalent. Overall, compared to the other three sex prediction tools, our proposed method is highly robust and shows better or similar performance in clustering females and males.

### Performance evaluation

The DNA methylation profiles of samples from training set and validation set are assessed by 450k array and EPIC array respectively. As we can see from the results (Fig. 2), the proposed model has correctly classified all samples in the two datasets, proving that the proposed classifier is highly robust and compatible with both platforms.

**Fig. 4:**
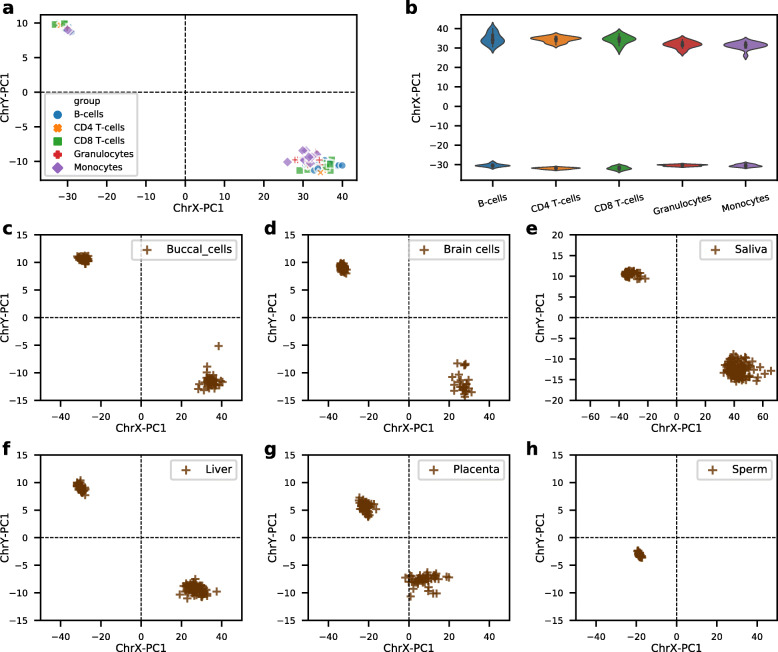
The sex classifier was evaluated across five blood cell types (**a** and **b**) and six other human tissues (**c**-**h**). **a** Scatter plot showing results from five blood cell types: B cells, CD4 T cells, CD8 T cells, monocytes and granulocytes. **b** On X chromosome, the five blood cell types showing similar results. **c** Buccal cells; **d** Brain cells; **e** Saliva; **f** Liver; **g** Placenta; **h** Sperms

The proposed sex classifier is trained and validated using whole blood samples. As whole blood is a heterogeneous collection of different cell types, to investigate whether our classifier is biased by blood cell types, we tested its performance on DNA methylation data derived from five purified blood cell types–B cells, CD4 T cells, CD8 T cells, monocytes and granulocytes from 28 individuals. As shown in Fig. 4a and b, all the five cell types are clustered into two sex groups and we could not find any or very minor differences between cell types. Collectively, these results suggest that the proposed sex classifier is robust to blood cell types.

Although blood is the most studied tissue in EWAS, there are also many DNA methylation studies that use samples from other types of human tissue. To evaluate our sex classifier’s range of application, we further tested its performance on several other most studied human tissues, including saliva, buccal cells, brain cells, liver, placenta, and sperm. Results from Fig. 4c to f demonstrate that the proposed classifier is robust in these vastly different types of tissues–saliva, buccal cells, brain cells, and liver. However, even though we can observe two clusters within the placenta samples, the female samples are more loosely distributed along the x-axis than that in other tissues, and all of them are more close to the zero point of x-axis, with several samples even have negative values (Fig. 3g).

Interestingly, all sperm samples were clustered into a single group by our sex classifier, located in the bottom left region (Fig. 3h). This area is typically recognised by our sex classifier as 45,XO. As sperm cells are a mixture of two types of haploid cells (23,X and 23,Y) this suggests that their methylation levels are lower on ChrY compared to other mature human tissues.

### Predicting sex chromosome aneuploidy

**Fig. 5:**
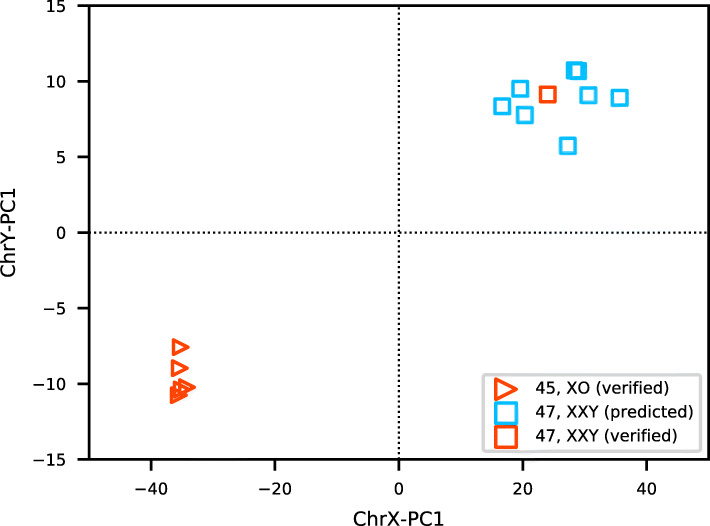
The proposed classifier is verified its ability to predict sex chromosome aneuploidy in five Turner syndrome samples and one Klinefelter syndrome case, it also predicted eight potential 47,XXY cases from GEO

DNA methylation has been an important way to study the various developmental symptoms caused by copy number aberrations of the sex chromosome [21]. Earlier, we proposed that our classifier can be applied to identify samples with abnormal sex chromosomes, including 45,XO and 47,XXY. To further validate its ability, we searched the public repositories for positive samples with clinical diagnosis. As a result of this, we obtained five cases (Table 2) diagnosed as Turner syndrome from two studies [22, 23]. As hoped, they are all clearly classified as 45,XO by our model (Fig. 5), proving our classifier’s ability to predict females with only one X chromosome.

**Table 2:** Samples with verified or suspect abnormal karyotypes from GEO

Sample ID	Karyotype	Verified karyotype?	Source tissue	Disease status	Reference
GSM1566904	45,XO	Yes	Peripheral Blood	Turner syndrome	[22]
GSM1566905	45,XO	Yes	Peripheral Blood	Turner syndrome	[22]
GSM1566906	45,XO	Yes	Peripheral Blood	Turner syndrome	[22]
GSM1566907	45,XO	Yes	Peripheral Blood	Turner syndrome	[22]
GSM1572595	45,XO	Yes	Whole Blood	Turner syndrome	[23]
3999215192_R06C02	47,XXY	Yes	Prefrontal cortex	Schizophrenia and Klinefelters syndrome	[24]
GSM3562874 (GSM3667736)^*^	47,XXY	No	Whole blood		[25]
GSM1649023	47,XXY	No	Whole blood		[26]
GSM1946555	47,XXY	No	Whole Blood	Post-traumatic stress disorder	[27]
GSM3662121	47,XXY	No	Blood	Lynch-like syndrome	NA
GSM1344329	47,XXY	No	Peripheral blood		[28]
GSM2336820	47,XXY	No	CD8+ T-cells	Ulcerative colitis	[29]
GSM3680912	47,XXY	No	Frontal cortex	Schizophrenia	[30]
GSM1496810	47,XXY	No	Frontal cortex	Schizophrenia	[31]

^*^GSM3562874 and GSM3667736 refer to the same case.

Viana et al. reported a male with schizophrenia carrying an extra X chromosome [24] which is also clearly classified as 47,XXY by our method (Fig. 5). Unfortunately, we did not find any publicly available DNA methylation samples from those diagnosed with Klinefelter syndrome. Unlike Turner syndrome, most patients with Klinefelter syndrome have only mild symptoms and are never diagnosed. It is interesting to check if there are any samples in GEO having a karyotype of 47,XXY but not linked to a diagnosis? By applying our classifier to scan the GEO datasets, we find a total of eight samples (Table 2) which are highly likely to be 47,XXY (Fig. 5). It should be noted that we only include these samples sourced from blood or brain cells related tissues and their DNA methylation level are assessed by 450K or EPIC arrays; we also do not include those samples which located near the boundaries which may be low-level sex chromosome mosaics (46,XX/47,XXY). It is interesting that two of the eight suspect abnormal samples were diagnosed with schizophrenia. Martin et al. found that Klinefelter patients have nearly a four times higher risk of schizophrenia [32], which may explain why we have predicted more 47,XXYs with schizophrenia. Studying the methylation patterns of these syndromes will provide more insights into these diseases.

## Discussion

There are two principal reasons to require a good and simple sex classifier based on methylation data. First, there are still many samples in GEO that do not have sex annotations, thus an accurate classifier can provide reliable sex information. Second, due to data entry errors, there are non-negligible proportions of mislabelled samples in the public database. A mismatch between reported sex and predicted sex would be a clear indication of a wrong annotation and introduces doubt on the accuracy of the rest of the phenotype information for that sample, hence it is reasonable to remove these mislabelled samples before downstream analyses. We would recommend sex checking to be a standard part of all DNA methylation QC pipelines. Here in this study, the proposed sex classifier is straightforward and the outcomes are highly intuitive.

In this study, we first obtained a group of significant sex-associated CpG sites. 90% of these located on the X chromosome are more methylated in females than that in males, this is mainly due to the effect of X-chromosome inactivation: one of the two X chromosomes in females is randomly chosen for inactivation (highly methylated) to balance the extra gene expression dosage [33, 34]. This also justified that our classifier was built on blood samples could work well across a wide range of other tissue types.

The proposed sex classifier shows robust performance across a wide range of tissue types despite it is built upon whole blood samples. We choose blood samples because they are easily accessible and are the most widely used tissue for measuring DNA methylation and have been adopted in most large cohort studies. However, whole blood is a heterogeneous collection of different cells, and their cell composition changes across age [35]. Different cell types can have distinct methylation profiles even though they share identical genetic makeup [36]. Here as our results have shown that the proposed model is not biased among different blood cell types; we also demonstrated the proposed classifier performs well across a wide range of human tissues, including saliva, buccal cells, brain cells, liver. These results suggest that our model is not driven by blood-specific sex differences, but it has captured the more general sex-associated differences across human tissues and cell types. However, we have also found some tissues such as placenta (Fig. 3h) showing an ambiguous boundary between the two sexes. Placenta is a fetal-maternal endocrine organ responsible for ensuring proper fetal development throughout pregnancy [37]. The fetal part of the placenta has the same genetic composition as fetus, whereas it exhibits apparent different DNA methylation patterns. Our results demonstrate placenta samples are less distinguishable between the two sex groups, showing both ChrX in female placentas and ChrY in male placentas are less methylated than that in other normal tissues. During the early development of human embryo, sperm cells are highly methylated and then become hypomethylated after fertilization [38]. Our results have shown that those sex-associated CpG on X chromosomes of sperm cells exhibited similar methylation patterns with other normal male tissues, however, the Y chromosomes are much less methylated. Collectively, our method can also be used to compare the methylation level of the two sex chromosomes in different tissues.

Our method can be readily applied to almost all DNA methylation datasets in GEO. Nearly a half of the DNA methylation datasets uploaded to GEO are not in IDAT format, which is prerequisite by using *minfi* and *ewastools*, many of these datasets only include intensity values of the methylated and unmethylated signals. Our sex classifier developed in this paper is based on beta values of those differently methylated CpG loci between the two sexes, users are only required to feed the whole beta value matrix, which can be easily computed from the signal intensity text files, to the ‘estimateSex’ function in *wateRmelon* to obtain final sex predictions.

The underlying mechanism of our sex classifier is very intuitive: females have higher levels of methylation on ChrX, on the contrary, males are less methylated on ChrX and show strong methylation signals on ChrY. We have also demonstrated that the proposed classifier can be applied on both 450K and EPIC arrays. Compared to signal density-based methods such as *minfi* and *ewastools*, the methylation ratio-based method from our sex classifier and *sEst* provide better separation between the two sexes (Fig. 4). In addition, both *minfi* and *ewastools* require at least one female and one male in the input samples to make correct sex predictions, however, our method and *sEst* do not have a such limitation. Lastly, our method has a much higher advantage over *sEst* on running speed and this is especially the case when applied to large sample size, for example, our method is more than four times faster than *sEst* when the number of input samples exceeds 1,000. Our speed advantage lies in that we saved the pre-trained weights for those sex-associated CpGs and only matrix multiplication is required to make sex classification, however, *sEst* requires to perform two seperate PCAs which are very time consuming.

We have provided a powerful tool that can identify sex chromosome aneuploidies (45,XO and 47,XXY) from DNA methylation data. This function has been verified in five Turner syndrome samples and one Klinefelter syndrome case, we should acknowledge that we need much more positive cases to testify its sensitivity and specificity. It is a pity that we did not find any DNA methylation samples labelled as Klinefelter syndrome in the public repositories. Nevertheless, we found eight cases in the GEO database with great potential to be 47,XXY by applying our classifier, with the knowledge that most patients with Klinefelter syndrome have only mild symptoms and are never diagnosed. Those eight suspect Klinefelter syndrome cases can be good candidates to study the various developmental symptoms caused by copy number aberrations of sex chromosomes.

## Conclusion

In this study, we constructed a very biological intuitive sex classifier, simply based on the most robust CpG sites on the sex chromosomes, which not only can be used for sex predictions but also applied to identify samples with sex chromosome aneuploidy. Our classifier has been integrated into the *wateRmelon* Bioconductor package, which is freely and easily accessible by calling the ‘estimateSex’ function.

## Methods

### Data collection and preprocess

We downloaded publicly available methylation datasets from GEO (https://www.ncbi.nlm.nih.gov/geo/), for those datasets which raw IDAT files were not available, such as GSE78874 and GSE137884, the intensity values of methylated and unmethylated signals were extracted from raw intensity text files. While for most of the datasets in which raw IDAT were provided, we used the function ‘iadd2’ from bigmelon package [39] to read and load intensity values from IDAT files. After that, beta values are calculated as:

$$\beta = \frac{M}{M + U + 100} $$

where *β* is beta value, *M* denotes methylated densities and *U* represents unmethylated densities. Beta values are ranged between 0 and 1, beta value close to 1 means high-level methylation and a near-zero beta value represents low level methylation. With manual inspection, those samples with apparent abnormal beta value density distributions were removed prior to downstream analysis. Also, those samples with more than 10% missing data were excluded.

There are 453,152 probes that exist in both 450k array and EPIC array, therefore, we only keep the shared 453,152 probes for downstream analysis. For each sample, the missing values of each probe were replaced by their corresponding means across all samples. Then, Z-score normalization was applied to each sample separately to reduce technical variance, which means all beta values were transformed to their Z-score values by subtracting the mean of all autosomal beta values and then divided by the standard deviation of all autosomal beta values within a sample. Z-score transformed beta values were used to construct PCA models and were used to make sex predictions.

### Model construction

GSE105018 was used to screen for sex-associated CpGs, it includes 1658 whole blood DNA methylation samples from participants in the Environmental Risk Longitudinal Twin Study, there are 826 female samples and 832 male samples in this dataset, with all participants aged at 18, among them, 1468 participants who were members of complete twin pairs (430 MZ pairs and 304 DZ pairs).

To identify sex-associated probes, T-test was applied to raw beta values of each of the 453,152 probes for the two sex groups, after Bonferroni multiple comparison correction, those probes with *p*-value less than 0.01 and absolute beta value difference between sexes greater than 0.2 were selected as significant sex-associated probes.

In order to have equal ratios of sexes, we randomly selected 800 females and 800 males from GSE105018, the Z-score transformed beta values of the identified sex-associated probes which mapped on sex chromosomes were used as input data. To be specific, the Z-score transformed beta values of the sex-associated probes which mapped on X chromosomes were processed by PCA, and the coefficients of the first principal component were used in the final model to distinguish whether a sample contains one copy X chromosome or two copy X chromosomes. Similarly, the Z-score transformed beta values of the sex-associated probes which mapped on Y chromosomes were processed by another PCA, and the coefficients of the result first principal component were used in the final model to distinguish whether a sample has Y chromosomes or not. As a result, the final model includes two sets of coefficients from two first principal components of two separate PCAs. Finally, the proposed sex classifier was tested by UKHLS dataset, with the labelled sexes as true sex annotations.

### Statistics analysis

All statistical analyses were conducted by Python (version 3.7.4, https://www.python.org/). T-tests were performed by using the function ‘stats.ttest_ind’ in the Scipy library [40]. The principal components analyses (PCAs) were performed by using the ‘decomposition.PCA’ function in Scikit-learn module [41].

**Table 3:** Summary of datasets used in this study

Dataset	Source	Platform	Number	Male/Female	Age(years)	Reference
GSE105018	Whole blood	450k	1658	832/826	18 - 18	[16]
UKHLS	Whole blood	EPIC	1175	489/686	28 - 98	[49]
GSE103541	Purified blood cells	EPIC	145	NA	NA	[42]
GSE137884	Buccal cells	450k	89	51/38	3 - 6	[43]
GSE112179	Brain cells	EPIC	100	75/25	23 - 77	[44]
GSE78874	Saliva	450k	259	146/113	36 - 88	[45]
GSE119100	Liver	EPIC	108	46/62	25 - 71	[46]
GSE100197	Placenta	450k	102	NA	NA	[47]
GSE64096	Sperms	450k	40	NA	NA	[48]
GSE51032	Buffy coat	450k	845	188/657	34 - 72	[20]

## Supplementary Information

**Additional file 1** Sex related differentially methylated CpGs. A list of the identified 4331 sex related differentially methylated CpGs on sex chromoseomes which are also used to construct the classifier.

## Data Availability

All the DNA methylation datasets except for the validation set analysed during the current study are publicly available and were obtained from the GEO public repository. The training set is from GSE105018 [16] which includes 832 male and 826 female whole blood samples, the validation set which includes 1175 whole blood samples is available from the European Genome-phenome Archive under accession EGAS00001002836 (https://www.ebi.ac.uk/ega/home). Other datasets: purified blood cell types (GSE103541 [42]), buccal cells (GSE137884 [43]), brain cells (GSE112179 [44]), saliva (GSE78874 [45]), liver (GSE119100 [46]), placenta (GSE100197 [47]), sperms (GSE64096 [48]). The one Klinefelter syndrome positive sample is available upon request. More details about these datasets are shown in Table 3.

## Abbreviations

GEO: Gene Expression Omnibus
450K: Infinium HumanMethylation450 BeadChip
EPIC: Infinium MethylationEPIC BeadChip
EWAS: Epigenome-wide Association Studies
PCA: Perform principal component analysis
ChrX: X chromosome
ChrY: Y chromosome
